# Allogeneic guinea pig mesenchymal stem cells ameliorate neurological changes in experimental colitis

**DOI:** 10.1186/s13287-015-0254-3

**Published:** 2015-12-30

**Authors:** Rhian Stavely, Ainsley M. Robinson, Sarah Miller, Richard Boyd, Samy Sakkal, Kulmira Nurgali

**Affiliations:** Centre for Chronic Disease, College of Health and Biomedicine, Western Centre for Health, Research and Education, Sunshine Hospital, 176 Furlong road, Melbourne, 3021 Victoria Australia; Department of Anatomy and Developmental Biology, Monash University, 19 Innovation Walk, Clayton, 3800 Victoria Australia

**Keywords:** Enteric neuropathy, Mesenchymal stem cells, Multipotent stromal cells, Colitis, Neuroprotection, Guinea pig, Bone marrow, Adipose tissue, Allogeneic, TGF-β

## Abstract

**Background:**

The use of mesenchymal stem cells (MSCs) to treat inflammatory bowel disease (IBD) is of great interest because of their immunomodulatory properties. Damage to the enteric nervous system (ENS) is implicated in IBD pathophysiology and disease progression. The most commonly used model to study inflammation-induced changes to the ENS is 2,4,6-trinitrobenzene-sulfonate acid (TNBS)-induced colitis in guinea pigs; however, no studies using guinea pig MSCs in colitis have been performed. This study aims to isolate and characterise guinea pig MSCs and then test their therapeutic potential for the treatment of enteric neuropathy associated with intestinal inflammation.

**Methods:**

MSCs from guinea pig bone marrow and adipose tissue were isolated and characterised *in vitro.* In *in vivo* experiments, guinea pigs received either TNBS for the induction of colitis or sham treatment by enema. MSCs were administered at a dose of 1 × 10^6^ cells via enema 3 h after the induction of colitis. Colon tissues were collected 24 and 72 h after TNBS administration to assess the level of inflammation and damage to the ENS. The secretion of transforming growth factor-β1 (TGF-β1) was analysed in MSC conditioned medium by flow cytometry.

**Results:**

Cells isolated from both sources were adherent to plastic, multipotent and expressed some human MSC surface markers. *In vitro* characterisation revealed distinct differences in growth kinetics, clonogenicity and cell morphology between MSC types. In an *in vivo* model of TNBS-induced colitis, guinea pig bone marrow MSCs were comparatively more efficacious than adipose tissue MSCs in attenuating weight loss, colonic tissue damage and leukocyte infiltration into the mucosa and myenteric plexus. MSCs from both sources were equally neuroprotective in the amelioration of enteric neuronal loss and changes to the neurochemical coding of neuronal subpopulations. MSCs from both sources secreted TGF-β1 which exerted neuroprotective effects *in vitro*.

**Conclusions:**

This study is the first evaluating the functional capacity of guinea pig bone marrow and adipose tissue-derived MSCs and providing evidence of their neuroprotective value in an animal model of colitis. *In vitro* characteristics of MSCs cannot be extrapolated to their therapeutic efficacy. TGF-β1 released by both types of MSCs might have contributed to the attenuation of enteric neuropathy associated with colitis.

## Background

Inflammatory bowel disease (IBD), comprising ulcerative colitis and Crohn’s disease, is a chronic debilitating disorder currently increasing in incidence and prevalence [1]. Patients experience severe manifestations, including bloody stool, persistent diarrhoea or constipation (or both), abdominal pain, ulcerations, fistulae, structuring and perianal fissures [2]. Current treatment options include anti-inflammatory drugs (aminosalicylates), corticosteroids, immunomodulators (thiopurines, methotrexate and cyclosporine) and biological agents (anti-tumour necrosis factor-alpha); these treatments either are toxic in the long term or frequently fail to induce and maintain remission [3]. Patients unresponsive to therapy require removal of inflamed bowel segments; incidentally, almost 30 % of patients with Crohn’s disease will undergo their first bowel resection surgery within 7 years of diagnosis and subsequently require repeated surgeries [4]. Therefore, investigations into alternative therapies for IBD are essential. Recently, there has been interest in the use of probiotics to treat IBD (e.g., Mutaflor) with positive results achieved in some clinical trials [5]. Using live cells to modify the inflammatory response, as opposed to conventional drugs, remains an intriguing prospect.

In the past decade, mesenchymal stem cells (MSCs), also known as multipotent stromal cells, have emerged as a clinically viable therapy for many diseases, including IBD [6–8]. MSCs are defined by their differentiation capacity, adherence to plastic in standard culture and expression of specific surface markers [9]. MSCs are easily isolated from adult tissue sources, including adipose tissue and bone marrow; they are highly proliferative and fibroblast-like in appearance and form monolayer colonies in culture [10–13]. Furthermore, MSCs can be successfully transplanted between individuals and across species as they have been shown to be immune-evasive [14, 15]. The presence of chemokine receptors on MSCs facilitates their migration toward inflammatory sites [16]. Once engrafted, MSCs suppress inflammation by immunomodulation via secretion of anti-inflammatory mediators [17]. MSCs can also exert regenerative effects through the secretion of pro-angiogenic and trophic factors which promote endogenous mechanisms of repair [18–20]. These properties make MSCs an attractive therapeutic option for IBD and have been widely studied in experimental colitis models and more recently in clinical trials for the treatment of Crohn’s disease in which autologous (host-donor, the same individual) and allogeneic (host-donor, the same species) MSC transplants have been performed [21–23]. MSCs have also been shown to be neuroprotective in clinical trials and in a variety of disease models, including multiple sclerosis, brain and spinal cord injury, stroke, peripheral nerve injury, amyotrophic lateral sclerosis and neurodegenerative diseases [24–35]. These studies provide the foundation for investigating the potential efficacy of MSC therapy for the treatment of enteric neuropathy associated with intestinal inflammation.

Damage to the enteric nervous system (ENS) correlates with persistent intestinal inflammation and gut dysfunction and may provide an avenue for intervention in the treatment of IBD [36–39]. The ENS functions via a network of neurons and glial cells throughout the length of the gastrointestinal tract. Neuronal bodies and glial cells reside within the ganglia comprising two major plexuses: submucosal and myenteric. Neurons of the submucosal plexus primarily regulate intestinal secretion and vasodilation, whereas neurons of the myenteric plexus coordinate muscular contractions [40]. Within the ganglia, individual neuronal subpopulations have specific functions and are classified by morphology, electrophysiological properties and their immunoreactivity to neurochemical markers associated with the production of different neurotransmitters. In the myenteric plexus, the two major subpopulations of muscle motor neurons and interneurons are cholinergic and nitrergic. These neurons are identified by the expression of choline acetyltransferase (ChAT) and neuronal nitric oxide synthase (nNOS), indicating the synthesis of acetylcholine and nitric oxide, respectively.

Intestinal inflammation is associated with neuronal death and axonal damage [41–44]. Additionally, variations to the neurochemical coding of enteric neurons have been reported in experimental colitis models and human IBD biopsies; these changes are not restricted to sites of active inflammation [42, 45–48]. Specifically, changes to cholinergic and nitrergic neuronal subpopulations have been reported to be implicated in intestinal dysmotility [49]. Leukocyte infiltration to the enteric plexuses is predictive of postoperative disease reoccurrence in patients with Crohn’s disease [50–52]. These observations suggest that neurological changes perpetuate inflammatory relapse and thus the ENS is a viable target for therapy.

Guinea pigs are the most common species used to study the functional and morphological properties of the ENS. The current knowledge of the neurochemical coding, morphological types and functional classification of enteric neurons was elucidated in guinea pigs [40, 53]. Since the ENS is embedded within the gastrointestinal wall, clean dissection and isolation of enteric ganglia are the main hurdles to study the ENS; these can be more readily achieved in tissues from guinea pigs compared with other animals. Electrophysiological, biophysical and molecular studies of different functional types of enteric neurons and their ion channels have been performed predominantly in guinea pigs [53–55].

Experimental models in guinea pigs have significantly contributed to the understanding of the pathophysiology for many human diseases, including cardiovascular, pulmonary, infectious, auditory and gestational disorders [56–61]. The 2,4,6-trinitrobenzene-sulfonic acid (TNBS)-induced model of colitis in guinea pigs has provided valuable data on functional, morphological and immunohistochemical changes of the ENS associated with intestinal inflammation [42, 43, 62–65]. The efficacy of xenogeneic (host-donor, different species) human MSC treatments has been tested in guinea pig models of osteoarthritis and radiation damage to the nasal mucosa [66, 67]. Our recent study in the guinea pig model of TNBS-induced colitis demonstrated the therapeutic potential of human MSCs for the treatment of inflammation-induced enteric neuropathy [68]. These studies may have benefited from the use of allogeneic MSCs to better replicate transplantation in a clinical setting. Given the value of guinea pig models in biomedical research, an investigation establishing the functional efficacy of allogeneic guinea pig MSCs is warranted. In this study, we present data on the isolation, *in vitro* characterisation and *in vivo* application of allogeneic MSCs for the treatment of enteric neuropathy associated with experimental colitis in guinea pigs.

## Methods

### Animals

Male and female Hartley guinea pigs weighing 140–280 g were received from the South Australian Health and Medical Research Institute. All guinea pigs were housed in a temperature-controlled environment with 12-h day/night cycles and had *ad libitum* access to food and water. All procedures were performed under approval of the Victoria University Animal Experimentation Ethics Committee and conducted in accordance with the Australian National Health and Medical Research Council Code of Practice for the Care and Use of Animals for Scientific Purposes.

### Isolation of MSCs from guinea pig adipose tissue

Visceral adipose tissue was obtained from guinea pigs. Tissues were collected in minimum essential medium with alpha modifications (α-MEM) (Gibco, part of Life Technologies, Melbourne, Australia) supplemented with 100 U/ml penicillin/streptomycin (Gibco). Samples were cut into 10-mm strips and incubated at 37 °C for 30 min in 5 ml of α-MEM with 100 U/ml penicillin/streptomycin and 25 μg/ml liberase™ (Roche, Basel, Switzerland). The adipose tissue was placed in C-tubes (Miltenyi Biotec, Bergisch Gladbach, Germany) and homogenised with a GentleMACS automated dissociator (Miltenyi Biotec) prior to and after an additional incubation step for 30 min at 37 °C. Enzymatic digestion was then inhibited by placing tubes on ice and diluting samples with α-MEM supplemented with penicillin/streptomycin. The connective tissue was removed via filtration at 40 μm. Samples were centrifuged at 500 *g* for 5 min, supernatant was removed and the pellet of cells was resuspended in 1 ml of expansion medium (α-MEM supplemented with 100 U/ml penicillin/streptomycin, 1 % glutaMAX (Gibco) and 16.5 % foetal bovine serum (mesenchymal stem cell-qualified; Gibco). Cells were seeded into culture flasks containing expansion medium which was replaced every 24 h for 3 days to rid cultures of non-adherent contaminating cells.

### Isolation of guinea pig bone marrow-derived MSCs

Femurs obtained from guinea pigs were transversely cut along the epiphysis, and the medullary cavity was flushed with expansion medium by using a 26-G needle to obtain a bone marrow suspension. To remove debris, the bone marrow suspension was filtered through a 40-μm Falcon cell strainer (In Vitro Technologies, Melbourne, Australia) before being seeded into culture flasks containing expansion medium. The medium was replaced every 24 h for 3 days.

### Cell culture and passaging

MSCs derived from guinea pig bone marrow (gpBM-MSCs) and adipose tissue (gpAT-MSCs) used in this study were cultured to the fourth passage for all subsequent experiments. Cells were plated at an initial density of 60 cells/cm^2^ and incubated in expansion medium which was replenished every 48–72 h for 10–14 days until the cells were 70–85 % confluent (maximum). MSCs were trypsinised and either reseeded for expansion or collected for *in vitro* experiments and *in vivo* treatment of guinea pigs.

### Surface marker expression

MSCs were immunolabelled as previously described [69] with CD29-Alexa Fluor 488 (clone TS2/16), CD34-phycoerythrin (clone 581), CD45-PerCPCy5.5 (clone H130), CD44-Brilliant Violet 421 (clone IM7), CD73-Brilliant Violet 421 (clone AD2) and CD90-Alexa Fluor 647 (clone 5E10) (1:100) (BioLegend, San Diego, CA, USA). Data were acquired on a BD FACSCanto II flow cytometer with FACSDiva version 6.1 software (BD Biosciences, Sydney, Australia). Unlabelled cells were incubated with 7-aminoactinomycin D (7-AAD) (1:20) (Life Technologies, Melbourne, Australia) for 1 minute before acquisition to determine the viability of the cell suspensions.

### Differentiation assay

The differentiation potential of MSCs was assessed by using the StemPro Adipogenesis, Osteogenesis and Chondrogenesis Differentiation Kits in accordance with the instructions of the manufacturer (Life Technologies). To detect adipogenesis, MSCs were fixed in 10 % neutral buffered formalin after 2 weeks in culture and lipid vacuoles were stained with Oil red O (Sigma-Aldrich, Sydney, Australia) in 60 % (vol/vol) isopropanol. Cells were then counterstained with haematoxylin. To detect osteogenesis, MSCs were fixed in 10 % neutral buffered formalin after 3 weeks in culture and calcium deposits were stained with 2 % (wt/vol) Alizarin red S (Sigma-Aldrich) in distilled water. To determine chondrogenic differentiation, micromass pellets were fixed in 10 % neutral buffered formalin after 2 weeks in culture and stained with Alcian blue 8GX (Sigma-Aldrich). Pellets were embedded in optimal cutting temperature (OCT) compound (Tissue-Tek; Sakura, Tokyo, Japan) and sectioned at 6 μm for viewing under light microscopy.

### Colony-forming unit-fibroblast assay

MSCs were seeded in 90-mm size petri dishes at low density (100 cells per dish). Expansion medium was changed every 3–4 days. After 2 weeks in culture, MSCs were fixed and stained with 0.5 % (wt/vol) crystal violet (Sigma-Aldrich) in methanol for 30 min before colonies containing more than 50 cells (colony-forming unit-fibroblast, or CFU-f) were counted under a dissection microscope.

### Growth kinetics

MSCs were cultured in triplicates and seeded at 60 cells/cm^2^ in 25-cm^2^ cell culture flasks containing 5 ml of expansion medium which was replaced every 48–72 h. Cells were trypsinised and counted with a haemocytometer at days 3, 7 and 14. The population doubling level (PDL) was calculated by using the formula PDL = (log^2^ [final number of cells]) − (log^2^ [initial cells seeded]) [70].

### Characterisation of MSC cell morphology

MSCs were seeded at 100 cells/cm^2^ in six-well plates and analysed after 48 h. MSCs were morphologically characterised into one of two categories defined by the presence of elongated cell bodies with long thin processes (spindle) or flat bodies with irregular processes (flat).

### Induction of colitis

To induce colitis, TNBS (Sigma-Aldrich) was dissolved in 30 % ethanol to a concentration of 30 mg/kg and administered intrarectally 7 cm proximal to the anus (total volume of 300 μl) by a lubricated silicone catheter [43]. Guinea pigs were anesthetised with isoflurane (1–4 % in O_2_) during the procedure and held at an inverted angle to prevent leakage. Sham-treated guinea pigs underwent the same procedure without administration of TNBS.

### Administration of MSCs

Guinea pigs were treated with MSCs 3 h after TNBS administration at the peak of tissue damage [71]. MSCs were administered by enema at a dose of 1 × 10^6^ cells in 300 μl of sterile phosphate-buffered saline (PBS). Guinea pigs were weighed and monitored daily following treatment. At 24 or 72 h after TNBS administration, animals were culled via stunning and exsanguination [63]. Segments of the distal colon were collected for histological and immunohistochemical studies.

### Tissue preparation

Colon tissues were cut along the mesenteric border, stretched and pinned flat with the mucosal side up for wholemount preparations. Tissue samples were fixed overnight at 4 °C in Zamboni’s fixative (2 % formaldehyde and 0.2 % picric acid) and subsequently washed in dimethyl sulfoxide (DMSO) (Sigma-Aldrich) (3 × 10 min) and PBS (3 × 10 min) to remove fixative. Samples for histology were fixed in 10 % buffered formalin solution and stored in 70 % ethanol until embedding.

### Immunohistochemistry

Immunohistochemistry was performed on wholemount preparations of the longitudinal muscle and myenteric plexus. The preparations were dissected by removing the mucosa, submucosa and circular muscle layers to expose the myenteric plexus. After 1-h incubation in 10 % normal donkey serum (Merck Millipore, Melbourne, Australia) at room temperature, wholemount preparations were incubated overnight at 4 °C with primary antibodies: mouse anti-Hu (clone 15A7.1) (1:500) (Merck Millipore), goat anti-nNOS (1:500) (Novus Biologicals, Littleton, CO, USA), goat anti-ChAT (1:500) (Merck Millipore), mouse anti-CD45 (clone IH-1) (1:200) (Abcam, Melbourne, Australia) and rabbit anti-protein gene product 9.5 (PGP9.5) (1:500) (Abcam). Tissues were washed (3 × 10 min PBS) and incubated for 2 h at room temperature with secondary antibodies: donkey anti-mouse Alexa Fluor 594 (1:200), donkey anti-goat FITC 488 (1:200), donkey anti-mouse FITC 488 (1:200) and donkey anti-rabbit Alexa Fluor 594 (1:200) (all from Jackson ImmunoResearch Laboratories, West Grove, PA, USA). After washing, tissues were mounted on glass slides with fluorescent mounting medium (Dako North America, Inc., Carpinteria, CA, USA). For cross-sections, tissues were frozen in OCT compound and sectioned at a thickness of 30 μm. Cross-sections were labelled with mouse anti-CD45 (1:200) followed by donkey anti-mouse FITC 488 (1:200) as described above.

### Histology

Tissues were embedded in paraffin and cut into 5-μm sections which were then deparaffinised, cleared, and rehydrated in graded ethanol. Cross-sections of the colon were stained with haematoxylin and eosin and mounted on glass slides with distrene plasticizer xylene (DPX) mountant. Gross morphological damage in cross-sections of the distal colon was assessed by histological grading of four parameters: mucosal flattening (0 = normal, 3 = severe flattening), occurrence of haemorrhagic sites (0 = none, 3 = frequent sites), loss of goblet cells (0 = normal, 3 = severe loss of cells) and variation of the circular muscle (0 = normal, 3 = considerable thickening of muscular layer) [62, 68].

### Flow cytometric analysis of transforming growth factor-β1

Conditioned media from MSC cultures as described above were collected at 48 h and analysed on the same day. Samples and cytokine standards with known concentrations were prepared in accordance with the instructions of the manufacturer by using the Human TGF-β1 Single Plex Flex Set (BD Biosciences). Data were acquired by using a BD FACSCanto II flow cytometer with FACSDiva version 6.1 software. FCS files were exported from FACSDiva and analysed with FCAP array version 3 software (BD Biosciences) to produce standard curves and measurements of TGF-β1 concentrations within the sample (in picograms per millilitre). At least 450 events were acquired per sample.

### Neuroprotective function of MSC-released TGF-β1 *in vitro*

Myenteric plexuses were isolated in accordance with a protocol described by Grundmann et al. [72]. Tissues were trypsinised for 10 min before cells were seeded into 96-well plates pre-coated with Matrigel (BD Biosciences) diluted 1:10 in medium. Myenteric plexuses were cultured for 7 days with the medium changed every second day. Cultures were subjected to lipopolysaccharide (LPS) (100 ng/ml; Sigma-Aldrich) in conjunction with gpBM-MSCs or gpAT-MSCs (5 × 10^4^ cells per well), the TGF-β receptor 1 (TGF-βR1) inhibitor SB431542 (10 μM; Sigma-Aldrich) or DMSO as a vehicle control. After 3 h, cultures were washed with PBS and fixed in 10 % neutral buffered formalin for 1 h. Cells were permeabilised with Triton X100 (Sigma-Aldrich) and incubated overnight at 4 °C with the pan-neuronal primary antibody chicken anti-microtubule-associated protein-2 (anti-MAP-2) (1:5000) followed by a 2-h incubation with the secondary antibody donkey anti-chicken Alexa Fluor 594 (1:200).

### Imaging

Confocal microscopy was performed by using an Eclipse Ti confocal laser scanning system (Nikon, Tokyo, Japan). Fluorophores were visualised by using a 488-nm excitation filter for Alexa 488 or FITC and a 559-nm excitation filter for Alexa 594. Z-series images were acquired at a nominal thickness of 0.5 μm (512 × 512 pixels). In wholemount preparations, the total numbers of myenteric neurons immunoreactive (IR) for Hu, nNOS, and ChAT as well as CD45-IR cells were counted within eight randomly captured images (total area size of 2 mm^2^) per preparation at a × 60 magnification. To evaluate CD45-IR in cross-sections, eight randomly captured images were analysed by using ImageJ software (National Institutes of Health, Bethesda, MD, USA). Images were converted from red, green, and blue (RGB) to grayscale 8 bit then to binary; these images were then analysed to obtain the total number of CD45 immunoreactive cells within the mucosa, submucosa and muscle layers in a 650-μm^2^ area (512 × 512 pixels). Chondrogenic pellets and haematoxylin and eosin-stained colon sections were visualised by using an Olympus BX53 microscope (Olympus, Melbourne, Australia), and images were captured with CellSens™ software (Olympus). Cellular imaging *in vitro* was performed on an Olympus IX81 inverted microscope using the same software. In primary neuronal cultures, images were acquired in a 9-mm^2^ area and were analysed by using ImageJ software. Images were converted from RGB to grayscale 8 bit then to binary. From these images, the percentage area of MAP-2 immunoreactivity was measured and presented relative to untreated primary neuronal cultures to determine neuronal loss.

### Statistical analysis

Data analysis was performed by using GraphPad Prism version 6 (Graphpad Software Inc., La Jolla, CA, USA). Data were analysed by using Student’s *t* test (two-tailed) and one- or two-way analysis of variance when appropriate for multiple group comparisons followed by Tukey’s and Sidak’s post hoc test. For all analysis, a *P* value of less than 0.05 was considered significant. All data were presented as mean ± standard error of the mean.

## Results

### *In vitro* validation of gpBM-MSCs and gpAT-MSCs and characterisation of their morphology and growth kinetics

**Fig. 1:**
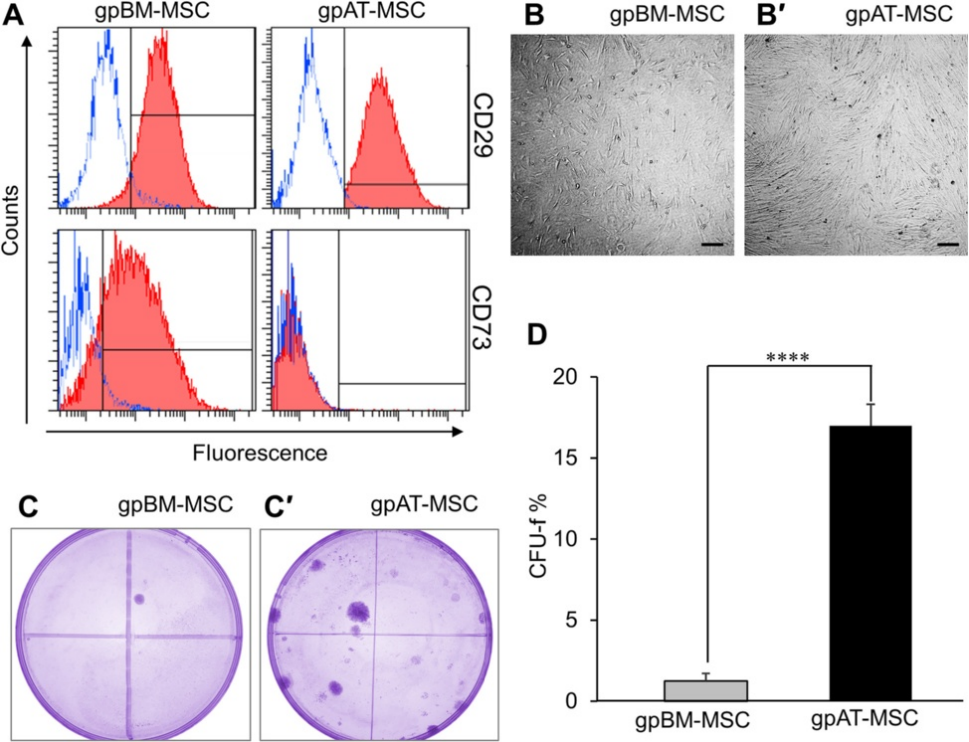
Immunophenotype and clonogenicity of guinea pig MSCs from bone marrow and adipose tissue. **a** GpBM-MSC and gpAT-MSCs were analysed for cell surface antigen expression of known positive MSC markers CD29 and CD73. Red closed histograms represent MSCs labelled with antibodies against the surface antigen indicated on the right-hand side of each row. Blue open histograms show isotype controls. GpBM-MSCs (**b**) and gpAT-MSCs (*b′*) adhered to plastic with a perceptible appearance typical of MSCs in culture. Scale bar = 200 μm. The clonogenicity of gpBM-MSCs (**c**) and gpAT-MSCs (*c′*) was determined by a colony-forming unit-fibroblast (CFU-f) assay (n = 4 independent cultures per group). **d** CFU-f counts were quantified as a percentage of the total viable cells seeded. ****P <0.0001. *gpAT-MSC* guinea pig adipose tissue-derived mesenchymal stem cell, *gpBM-MSC* guinea pig bone marrow-derived mesenchymal stem cell, *MSC* mesenchymal stem cell

To examine the immunophenotype of guinea pig MSCs, cells were analysed by flow cytometry for the presence of positive MSC markers CD29, CD44, CD73 and CD90 and negative MSC markers CD34 (hematopoietic progenitors) and CD45 (leukocyte common antigen). Positive expression of CD29 was observed in both gpBM-MSCs (95.1 %) and gpAT-MSCs (97.6 %). There was differential expression of the CD73 antigen such that 85.0 % of gpBM-MSCs expressed the antigen compared with negligible expression in gpAT-MSCs (0.2 %) (Fig. 1a). Negligible expression of the remaining surface markers was observed in both cell lines (gpBM-MSC: CD44, 1.9 %; CD90, 1.5 %; CD34, 4.4 %; CD45, 5.5 %. gpAT-MSC: CD44, 0.2 %; CD90, 0.2 %; CD34, 0.2 %; CD45, 0.7 %; data not shown).

**Fig. 2:**
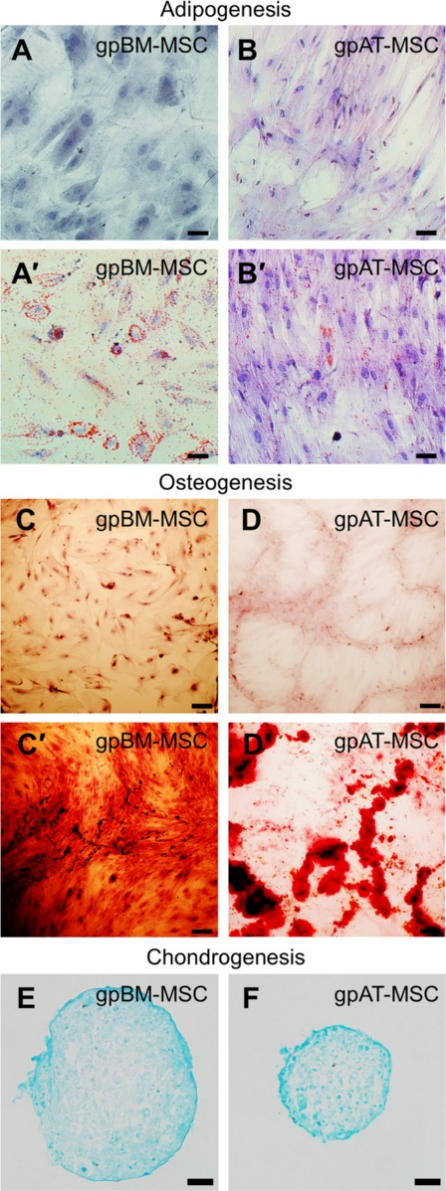
Differentiation potential of guinea pig MSCs. GpBM-MSCs and gpAT-MSCs cultured without (**a**, **b**) and with (*a′*, *b′*) adipogenesis differentiation medium for 14 days and stained with Oil red O. Scale bar = 50 μm. GpBM-MSCs and gpAT-MSCs cultured without (**c**, **d**) and with (*c′*, *d′*) osteogenesis differentiation medium for 21 days and stained with Alizarin red S. Scale bar = 200 μm. Alcian blue stained cross-sections of chondrogenic pellets formed by gpBM-MSCs (**e**) and gpAT-MSCs (**f**) after 14 days in chondrogenic differentiation medium. Scale bar = 50 μm. *gpAT-MSC* guinea pig adipose tissue-derived mesenchymal stem cell, *gpBM-MSC* guinea pig bone marrow-derived mesenchymal stem cell, *MSC* mesenchymal stem cell

Both bone marrow- and adipose tissue-derived MSCs grew in monolayer culture, adhered to plastic, proliferated and were typical of MSC appearance (Fig. 1b, b′). The clonogenicity of MSCs was compared via a CFU-f assay. The yield of colony-forming units (>50 cells per colony [73]) of gpAT-MSCs (17.0 ± 1.3 %) was higher than that of gpBM-MSCs (1.3 ± 0.5 %, *P <*0.0001) after 2 weeks in culture (Fig. 1c, c′, d, n = 4 independent cultures per group). To assess the multipotent potential of MSCs, cells were cultured in specialised media to induce adipogenic, osteogenic and chondrogenic differentiation. Lipid vacuoles were present in MSCs cultured in adipogenesis differentiation medium when stained with Oil red O indicative of successful differentiation into adipocytes (Fig. 2a–b′). MSCs cultured in osteogenesis differentiation medium were stained with Alizarin red S, confirming successful differentiation into osteocytes (Fig. 2c–d′). MSCs seeded as micromass cultures only formed pellets in chondrogenesis differentiation medium. Chondrogenic differentiation was confirmed in the pellets by Alcian blue staining of cross-sections (Fig. 2e, f).

**Fig. 3:**
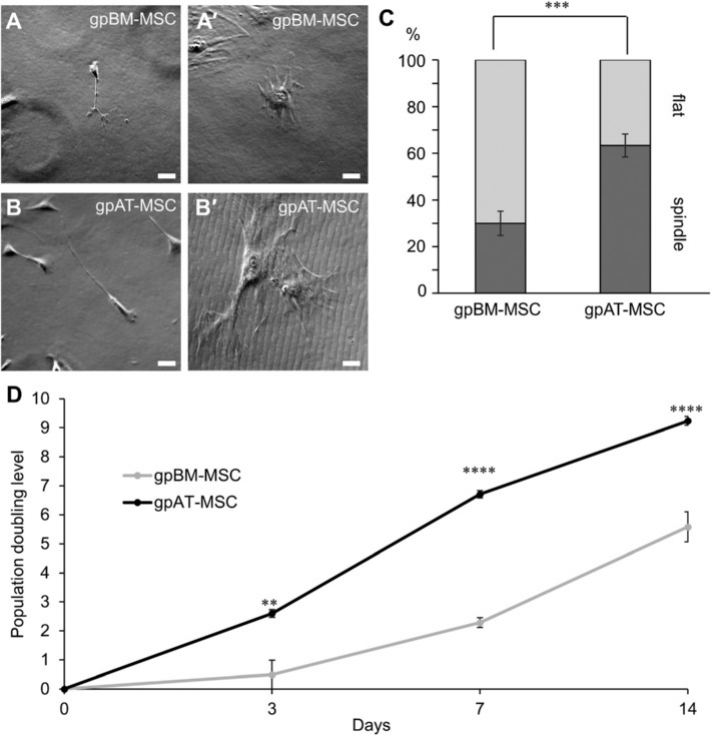
*In vitro* morphology and growth kinetics of guinea pig MSCs. **a**–*b′* Distinct morphological subpopulations were exhibited by gpBM-MSCs (**a**, *a′*) and gpAT-MSCs (**b**, *b′*) in culture. MSC morphology was defined according to the presence of long thin spindles (‘spindle’: **a**, **b**) or flat cells with atypical processes (‘flat’: *a′*, *b′*) (scale bar = 50 μm). **c** Quantitative analysis of MSC morphological types. Data are expressed as a percentage of the total cell number in each population (n = 6 independent cultures per group). **d** The population doubling level of proliferating MSCs was recorded at 3, 7 and 14 days after seeding (n = 3 independent cultures per group per time point). **P <0.01, ***P <0.001, ****P <0.0001. *gpAT-MSC* guinea pig adipose tissue-derived mesenchymal stem cell, *gpBM-MSC* guinea pig bone marrow-derived mesenchymal stem cell, *MSC* mesenchymal stem cell

MSCs were categorised and quantified according to the shape of their cell bodies (Fig. 3a–b′). Both gpBM-MSCs and gpAT-MSCs exhibited two morphological types: long thin ‘spindle’ (Fig. 3a, b) and ‘flat’ with irregular processes (Fig. 3a′, b′). Populations of cells with a ‘spindle’ morphology were higher in gpAT-MSCs (63.3 ± 4.9 %) than gpBM-MSCs (30.0 ± 5.2 %, *P <*0.001, n = 6 independent cultures per group; Fig. 3c). Inversely, gpBM-MSC populations had a higher proportion of cells with a ‘flat’ morphology (70.0 ± 5.2 %) in comparison with gpAT-MSCs (36.7 ± 4.9 %, *P <*0.001). The proliferation of MSCs was quantified over 14 days in culture and presented as the population doubling level (PDL) (Fig. 3d). The PDL of gpAT-MSCs was higher than that of gpBM-MSCs at 3 days (2.6 ± 0.1 versus 0.5 ± 0.5, *P <*0.01), 7 days (6.7 ± 0.1 versus 2.3 ± 0.2, *P <*0.0001) and 14 days (9.2 ± 0.2 versus 5.6 ± 0.5, *P <*0.0001) in culture (n = 3 independent cultures per group per time point).

### GpBM-MSCs are more efficacious than gpAT-MSCs in ameliorating histological damage and weight loss associated with TNBS-induced colitis

**Fig. 4:**
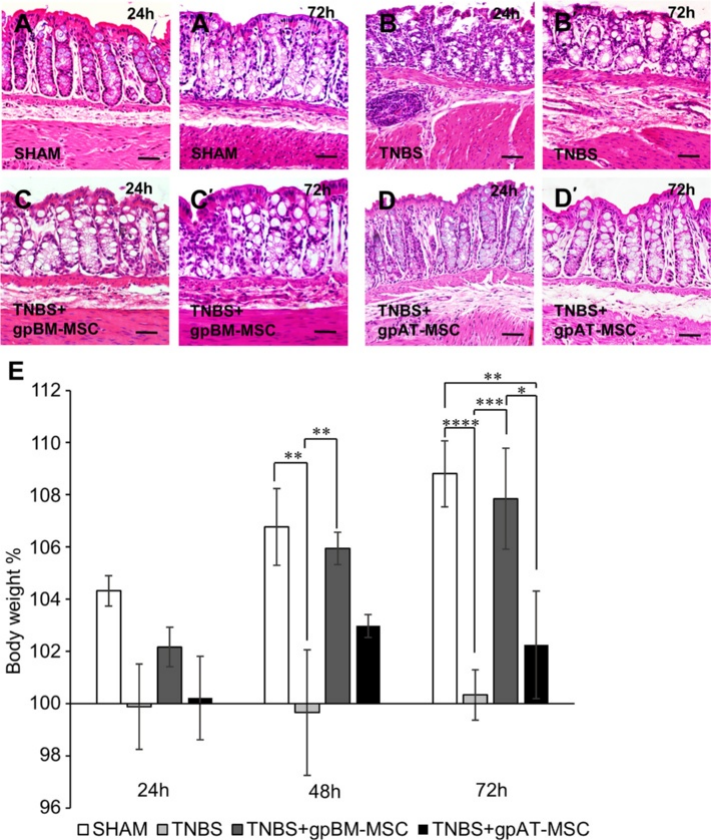
Effects of guinea pig MSC treatments on histological changes and body weight in colitis. Colonic structure was assessed via haematoxylin-and-eosin staining of cross-sections from tissues collected at 24 h (**a**–**d**) and 72 h (*a′*-*d′*) after TNBS administration. Scale bar = 50 μm. **e** Body weight was recorded at 24, 48 and 72 h after TNBS administration and is expressed as the change from baseline measurements. *P <0.05, **P <0.01, ***P <0.001, ****P <0.0001, significantly different between groups; ^†††^ *P <*0.001, ^††††^ *P <*0.0001, significantly different from baseline weight within groups, n = 4 animals per group per time point. *gpAT-MSC* guinea pig adipose tissue-derived mesenchymal stem cell, *gpBM-MSC* guinea pig bone marrow-derived mesenchymal stem cell, *MSC* mesenchymal stem cell, *TNBS* 2,4,6-trinitrobenzene sulfonic acid

Gross morphological damage was not observed in haematoxylin and eosin-stained cross-sections from sham-treated guinea pigs (histological score = 0; Fig. 4a, a′). Mucosal flattening, haemorrhagic sites, loss of goblet cells and altered presentation of the circular muscle layer denoting histological score of 2–3 were observed at 24 and 72 h following induction of colitis (Fig. 4b, b′). Minimal evidence of mucosal damage or disruption to the colonic architecture was observed in sections of the colon from gpBM-MSC-treated animals at both 24 and 72 h after TNBS administration (histological score = 0–1; Fig. 4c, c′). Structural changes in crypt architecture, mild mucosal damage and oedema in the submucosal layer were still present in colon sections from gpAT-MSC-treated animals (histological score = 1–2; Fig. 4d, d′).

The body weight of guinea pigs is indicative of overall disease progression. Guinea pig body weight was recorded at 24, 48 and 72 h after treatment (Fig. 4e and Table 1, n = 4 animals per group per time point). No statistical differences were observed in weight change at 24 h between all groups. The body weight of TNBS-administered animals was lower compared with sham (48 h, *P <*0.01 and 72 h, *P <*0.0001) and animals treated with gpBM-MSCs (48 h, *P <*0.01 and 72 h, *P <*0.001) but not gpAT-MSCs (48 h, *P =* 0.3 and 72 h, *P =* 0.7). By 72 h, gpBM-MSCs were more effective than gpAT-MSCs in promoting weight gain (*P <*0.05). At the same time point, only sham and gpBM-MSC groups had gained weight (both *P <*0.0001) which was not observed in TNBS (*P =* 1.0) or gpAT-MSC (*P =* 0.6) groups.

**Table 1:** Effects of allogeneic mesenchymal stem cells derived from guinea pigs on body weight (percentage) in TNBS-induced colitis

	Sham	TNBS	TNBS + gpBM-MSC	TNBS + gpAT-MSC
24 h	104.3 ± 0.6	99.9 ± 1.6	102.2 ± 0.8	100.2 ± 1.6
48 h	106.8 ± 1.5	100.4 ± 2.4††	105.9 ± 0.6**	103.0 ± 0.4
72 h	108.8 ± 1.3	100.3 ± 1.0††††	107.8 ± 1.9***	102.3 ± 2.1‡ ††

*TNBS* 2,4,6-trinitrobenzene sulfonic acid, *gpBM-MSC* guinea pig bone marrow-derived mesenchymal stem cell, *gpAT-MSC* guinea pig adipose tissue-derived mesenchymal stem cell. **P <0.01, ***P <0.001, significantly different from TNBS; ††*P <*0.01, ††††*P <*0.0001 significantly different from sham; ‡*P <*0.05, significantly different from gpBM-MSC

### GpBM-MSCs are more efficient in attenuating leukocyte infiltration to the mucosa and myenteric ganglia compared with gpAT-MSCs

**Fig. 5:**
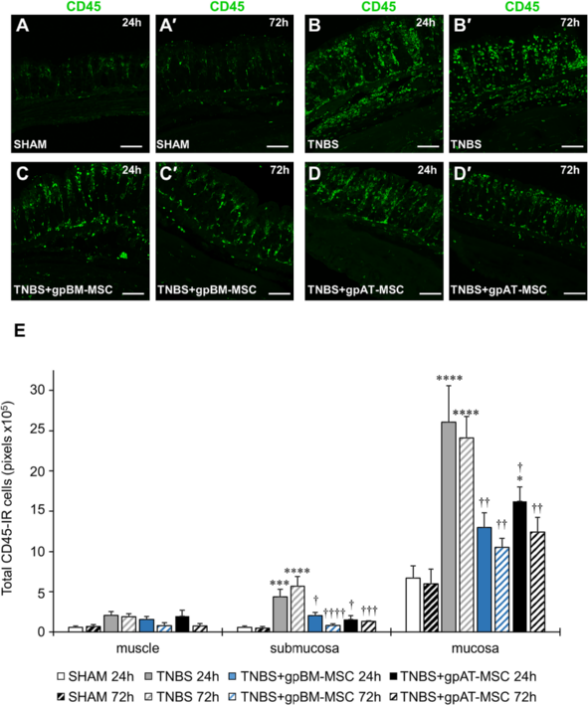
Effects of guinea pig MSC treatments on leukocyte infiltration in the colonic wall. **a**–*d′* CD45-IR leukocytes were visualised within the mucosa, submucosa and muscle layers of the colon. Cross-sections from guinea pig colon collected at 24 h (**a**–**d**) and 72 h (*a′*–*d′*) after treatment. Scale bar = 100 μm. **e** All of the CD45-IR cells were quantified in the mucosa, submucosa and muscle layers within a 650-μm^2^ area of the colon. *P <0.05, ***P <0.001, ****P <0.0001, significantly different from sham; †*P <*0.05, ††*P <*0.01, †††*P <*0.001, ††††*P <*0.0001, significantly different from TNBS; n = 4 animals per group per time point. *gpAT-MSC* guinea pig adipose tissue-derived mesenchymal stem cell, *gpBM-MSC* guinea pig bone marrow-derived mesenchymal stem cell, *MSC* mesenchymal stem cell, *TNBS* 2,4,6-trinitrobenzene sulfonic acid

**Fig. 6:**
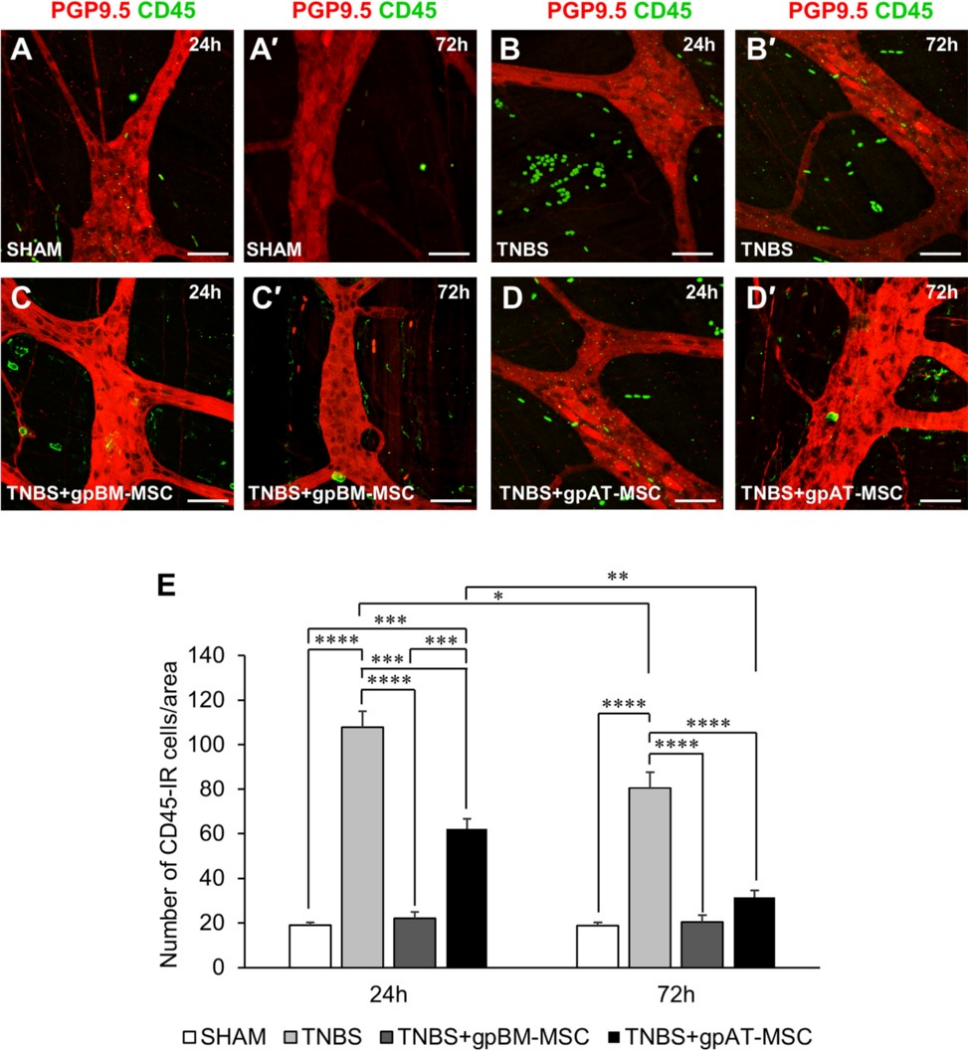
Effects of treatments with guinea pig MSCs on leukocyte infiltration to the myenteric plexus. **a**–*d′* CD45-IR leukocytes (*green*) were visualised on the level of myenteric neurons labelled with anti-PGP9.5 (*red*) by confocal microscopy. Wholemounts of the myenteric plexus from guinea pig colon preparations collected at 24 h (**a**–**d**) and 72 h (*a′*-*d′*) after treatment. Scale bar = 50 μm. **e** CD45-IR leukocytes were quantified in a 2-mm^2^ area of the myenteric plexus in the colon. **P <0.01, ***P <0.001, ****P <0.0001, n = 4 animals per group per time point. *gpAT-MSC* guinea pig adipose tissue-derived mesenchymal stem cell, *gpBM-MSC* guinea pig bone marrow-derived mesenchymal stem cell, *MSC* mesenchymal stem cell, *PGP9.5* protein gene product 9.5

Leukocyte infiltration to the mucosa, submucosa and muscle layers was quantified in cross-sections of the guinea pig colon by using an antibody to the pan-leukocyte marker CD45 (Fig. 5a–d′, n = 4 animals per group per time point). Administration of TNBS resulted in an increase in leukocytes at 24 and 72 h within the mucosa (24 and 72 h, *P <*0.0001) and submucosa (24 h, *P <*0.001 and 72 h, *P <*0.0001) (Table 2 and Fig. 5e). Greater leukocyte numbers in TNBS groups were attenuated by treatments with both gpBM-MSCs and gpAT-MSCs at 24 and 72 h in the mucosa (gpBM-MSC: 24 and 72 h, *P <*0.01; gpAT-MSCs: 24 h, *P <*0.05 and 72 h, *P <*0.01) and submucosa (gpBM-MSC: 24 h, *P <*0.05 and 72 h, *P <*0.0001; gpAT-MSCs: 24 h, *P <*0.05 and 72 h, *P <*0.001). However, at 24 h, leukocyte numbers were higher in the mucosa of gpAT-MSC-treated animals compared with shams (*P <*0.05). No differences in leukocyte infiltration were observed in cross-sections of the muscle layer. Leukocyte infiltration to the level of the myenteric plexus was quantified in wholemount preparations of the guinea pig colon (Fig. 6a–d′, n = 4 animals per group per time point). At 24 h, leukocyte numbers were elevated in animals administered with TNBS only (107.8 ± 7.2 cells per area) compared with shams (19 ± 1.2 cells per area, *P <*0.0001; Fig. 6e). The infiltration of leukocytes was attenuated by treatment with both gpBM-MSCs (22 ± 2.9 cells per area, *P <*0.0001) and gpAT-MSCs (62 ± 4.6 cells per area, *P <*0.001). However, in the latter, leukocytes were still elevated compared with sham (*P <*0.001) and gpBM-MSC (*P <*0.001) treated animals. At 72 h, the number of leukocytes remained elevated in TNBS-administered animals (80.5 ± 7.3 cells per area) compared with sham (18.8 ± 1.4 cells per area, *P <*0.0001) which was decreased by both gpBM-MSC (20.5 ± 3.1 cells per area, *P <*0.0001) and gpAT-MSC (31.5 ± 3.2 cells per area, *P <*0.0001) treatments to levels comparable to those of the sham group. Between 24 and 72 h, there was no change in the levels of leukocytes in sham (*P >*0.99) and gpBM-MSC-treated (*P >*0.99) groups. Conversely, a decrease in the number of leukocytes was observed in TNBS (*P <*0.05) and gpAT-MSC-treated (*P <*0.01) groups.

**Table 2:** Effects of *in vivo* treatment with allogeneic mesenchymal stem cells derived from guinea pigs on CD45 immunoreactive leukocytes (pixels × 10^5^) in cross-sections of the colon

	Sham	TNBS	TNBS + gpBM-MSC	TNBS + gpAT-MSC
	Mucosa			
24 h	6.71 ± 1.52	26.09 ± 4.47****	12.97 ± 1.83††	16.22 ± 1.80†*
72 h	6.00 ± 1.82	24.13 ± 2.65****	10.54 ± 1.11††	12.44 ± 1.78††
	Submucosa			
24 h	0.59 ± 0.17	4.38 ± 0.92***	2.02 ± 0.41†	1.56 ± 0.46†
72 h	0.48 ± 0.20	5.69 ± 1.22****	0.82 ± 0.19††††	1.31 ± 0.10†††
	Muscle			
24 h	0.57 ± 0.19	2.07 ± 0.48	1.55 ± 0.38	1.96 ± 0.74
72 h	0.68 ± 0.22	1.91 ± 0.36	0.78 ± 0.39	0.76 ± 0.30

*TNBS* 2,4,6-trinitrobenzene sulfonic acid, *gpBM-MSC* guinea pig bone marrow-derived mesenchymal stem cell, *gpAT-MSC* guinea pig adipose tissue-derived mesenchymal stem cell. †*P <*0.05, ††*P <*0.01, †††*P <*0.001, ††††*P <*0.0001, significantly different from TNBS; *P <0.05, ***P <0.001, ****P <0.0001, significantly different from sham; n = 4 animals per group per time point

### GpBM-MSCs and gpAT-MSCs have comparable efficacy for attenuating inflammation-induced enteric neuropathy

**Fig. 7:**
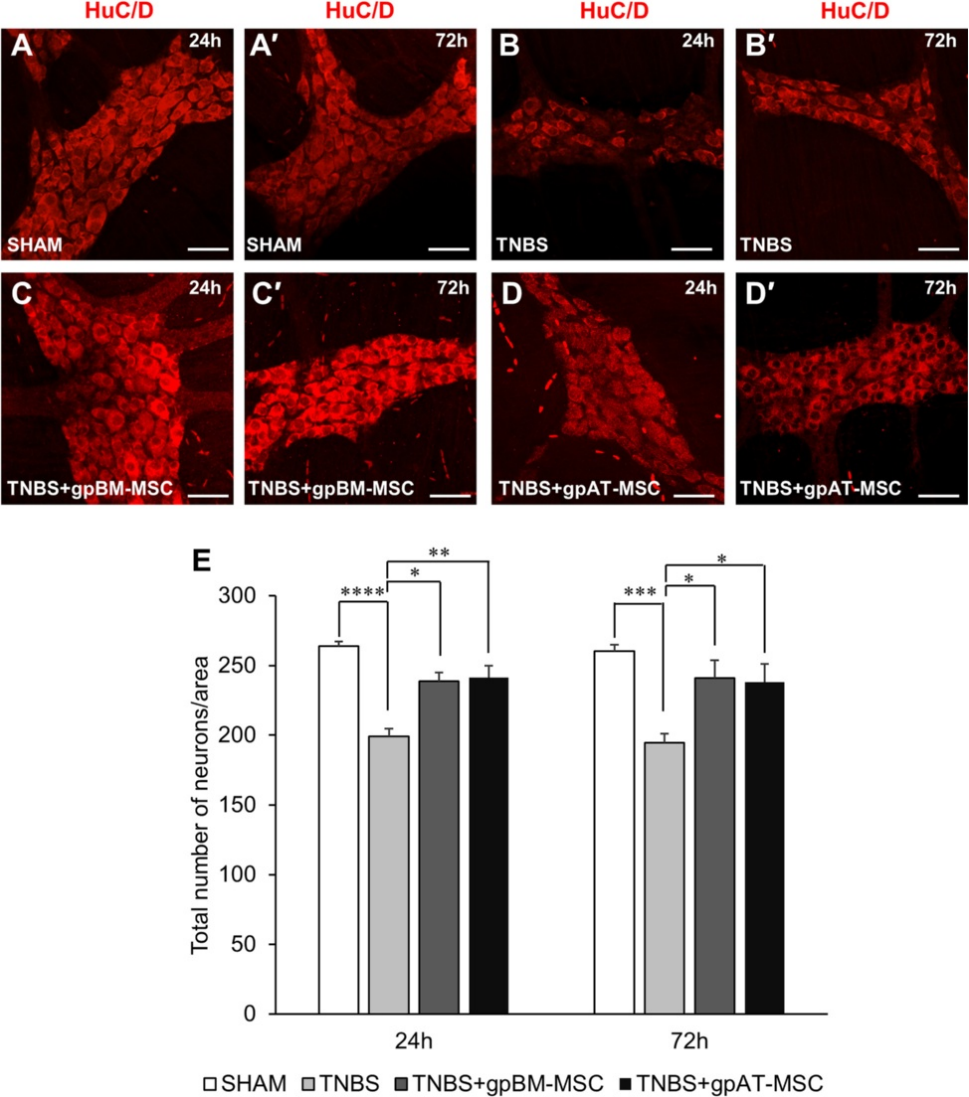
Effects of guinea pig MSCs on the total number of myenteric neurons. **a**–*d′* Neuronal cell bodies in the myenteric plexus were labelled with the pan-neuronal marker anti-HuC/D antibody at 24 h **a**–**d** and 72 h (*a′*-*d′*) after treatment. Scale bar = 50 μm. **e** The total number of neuronal bodies was quantified within a 2-mm^2^ area of the myenteric plexus. *P <0.05, **P <0.01, ***P <0.001, ****P <0.0001, n = 4 animals per group per time point. *gpAT-MSC* guinea pig adipose tissue-derived mesenchymal stem cell, *gpBM-MSC* guinea pig bone marrow-derived mesenchymal stem cell, *MSC* mesenchymal stem cell, *TNBS* 2,4,6-Trinitrobenzene sulfonic acid

To assess the effect of MSC treatments on the total number of myenteric neurons, anti-HuC/D antibody was used as a pan-neuronal marker in wholemount preparations of the guinea pig colon and quantified per 2-mm^2^ area (Fig. 7a–d′, n = 4 animals per group per time point). The number of neurons in the myenteric plexus was reduced after TNBS administration compared with sham treatment at both 24 h (*P <*0.0001) and 72 h (*P <*0.001; Table 3 and Fig. 7e). The loss of myenteric neurons was alleviated by treatment with both gpBM-MSCs (24 and 72 h: *P <*0.05) and gpAT-MSCs (24 h: *P <*0.01 and 72 h: *P <*0.05).

**Table 3:** Effects of allogeneic mesenchymal stem cells derived from guinea pigs on myenteric neurons in TNBS-induced colitis

	Sham	TNBS	TNBS + gpBM-MSC	TNBS + gpAT-MSC
	Total number of myenteric neurons/2 mm^2^			
24 h	262.6 ± 2.9	204.6 ± 6.9††††	238.8 ± 6.2*	241.3 ± 8.6**
72 h	261.8 ± 3.9	203.4 ± 4.2†††	241.0 ± 12.7*	238.0 ± 13.2*
	Total number of nNOS-IR neurons/2 mm^2^			
24 h	51.8 ± 1.5	68.8 ± 3.9††	58.5 ± 4.3*	55.5 ± 4.4**
72 h	53.0 ± 1.0	67.6 ± 3.8†	56.0 ± 5.5*	55.8 ± 1.8**
	Proportion of nNOS-IR neurons/2 mm^2^, %			
24 h	19.8 ± 0.8	33.8 ± 2.4†††	24.6 ± 2.2**	23.0 ± 1.7***
72 h	20.3 ± 0.3	33.3 ± 1.9†††	23.3 ± 2.0**	23.6 ± 1.3**
	Total number of ChAT-IR neurons/2 mm^2^			
24 h	159.6 ± 3.9	111.4 ± 4.0††††	134.3 ± 5.7†**	135.3 ± 5.7†**
72 h	157.4 ± 4.5	110.2 ± 2.2††††	137.8 ± 1.5†**	135.3 ± 2.5†**
	Proportion of ChAT-IR neurons/2 mm^2^, %			
24 h	60.8 ± 1.6	54.6 ± 2.3	56.7 ± 4.1	56.0 ± 1.3
72 h	60.1 ± 1.2	54.3 ± 2.0	57.6 ± 3.0	57.2 ± 2.1

*TNBS* 2,4,6-trinitrobenzene sulfonic acid, *gpBM-MSC* guinea pig bone marrow-derived mesenchymal stem cell, *gpAT-MSC* guinea pig adipose tissue-derived mesenchymal stem cell, *nNOS* neuronal nitric oxide synthase, *ChAT* choline acetyltransferase, *IR* immunoreactive. †*P <*0.05, ††*P <*0.01, †††*P <*0.001, ††††*P <*0.0001, significantly different from sham; *P <0.05, **P <0.01, ***P <0.001, significantly different from TNBS

**Fig. 8:**
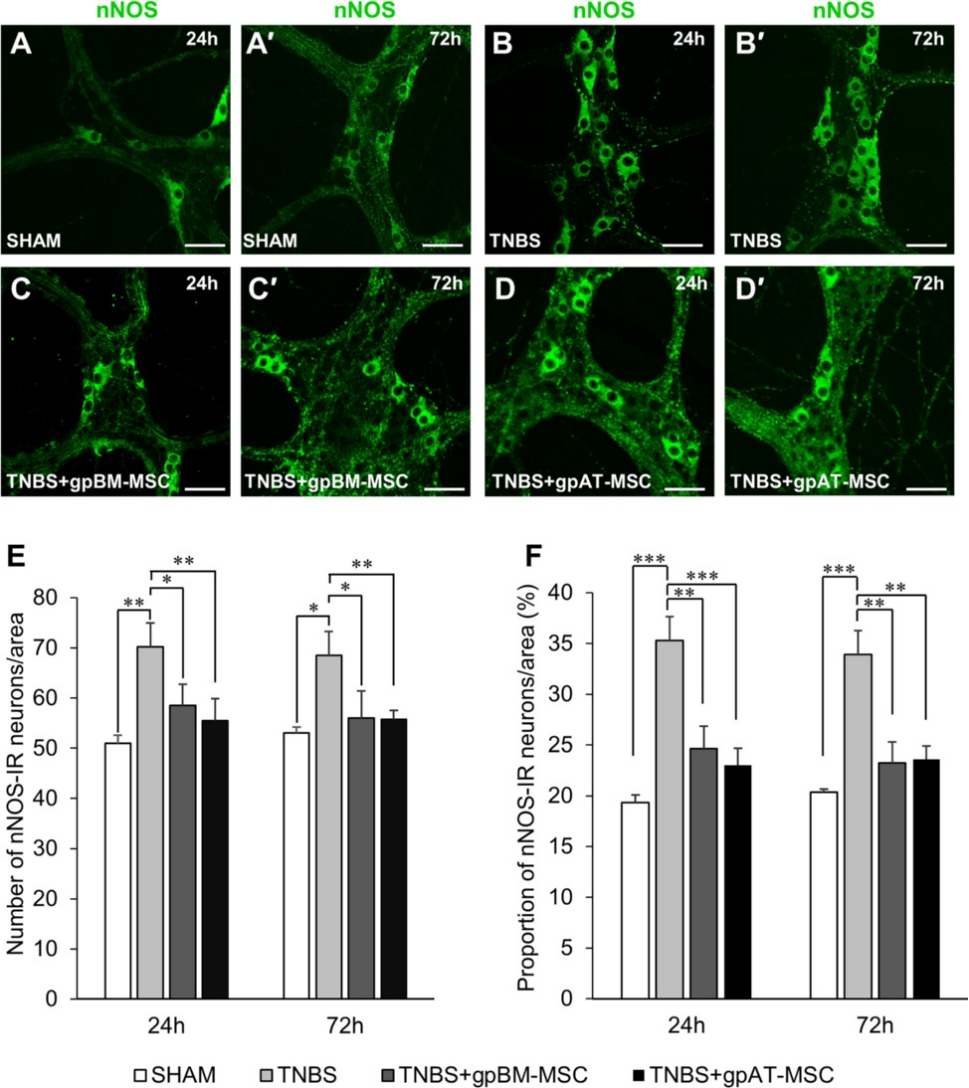
Effects of guinea pig MSCs on nitrergic myenteric neurons. **a**–*d′* Nitrergic (nNOS-IR) neurons were visualised in the myenteric plexus at 24 h (**a**–**d**) and 72 h (*a′*–*d′*). Scale bar = 50 μm. The total number of nNOS-IR neurons (**e**) and the proportion of nNOS-IR neurons to the total number of HuC/D-IR neurons (**f**) were quantified within a 2-mm^2^ area of the myenteric plexus in the guinea pig colon. *P <0.05, **P <0.01, ***P <0.001, n = 4 animals per group per time point. *gpAT-MSC* guinea pig adipose tissue-derived mesenchymal stem cell, *gpBM-MSC* guinea pig bone marrow-derived mesenchymal stem cell, *MSC* mesenchymal stem cell, *TNBS* 2,4,6-Trinitrobenzene sulfonic acid

The two major subpopulations of neurons in the myenteric plexus, inhibitory and excitatory muscle motor and interneurons, were investigated [74]. Inhibitory neurons were labelled with anti-nNOS antibody (Fig. 8a–d′), and the total number of nNOS-IR neurons was quantified per 2-mm^2^ area (Fig. 8e and Table 3, n = 4 animals per group per time point). The number of nNOS-IR neurons was increased in the myenteric plexus from TNBS groups compared with sham at 24 h (*P <*0.01) and 72 h (*P <*0.05). The proportion of nNOS-IR neurons to the total number of Hu-IR neurons was increased in TNBS groups at both time points compared with sham (24 and 72 h, *P <*0.001) (Fig. 8f, n = 4 animals per group per time point). Both MSC treatments ameliorated the increase in the total number (gpBM-MSC: 24 and 72 h, *P <*0.05 and gpAT-MSC: 24 and 72 h, *P <*0.01) and proportion of nNOS-IR neurons at all time points (gpBM-MSC: 24 and 72 h, *P <*0.01; gpAT-MSCs: 24 h, *P <*0.001 and 72 h, *P <*0.01) (Fig. 8e, f and Table 3).

**Fig. 9:**
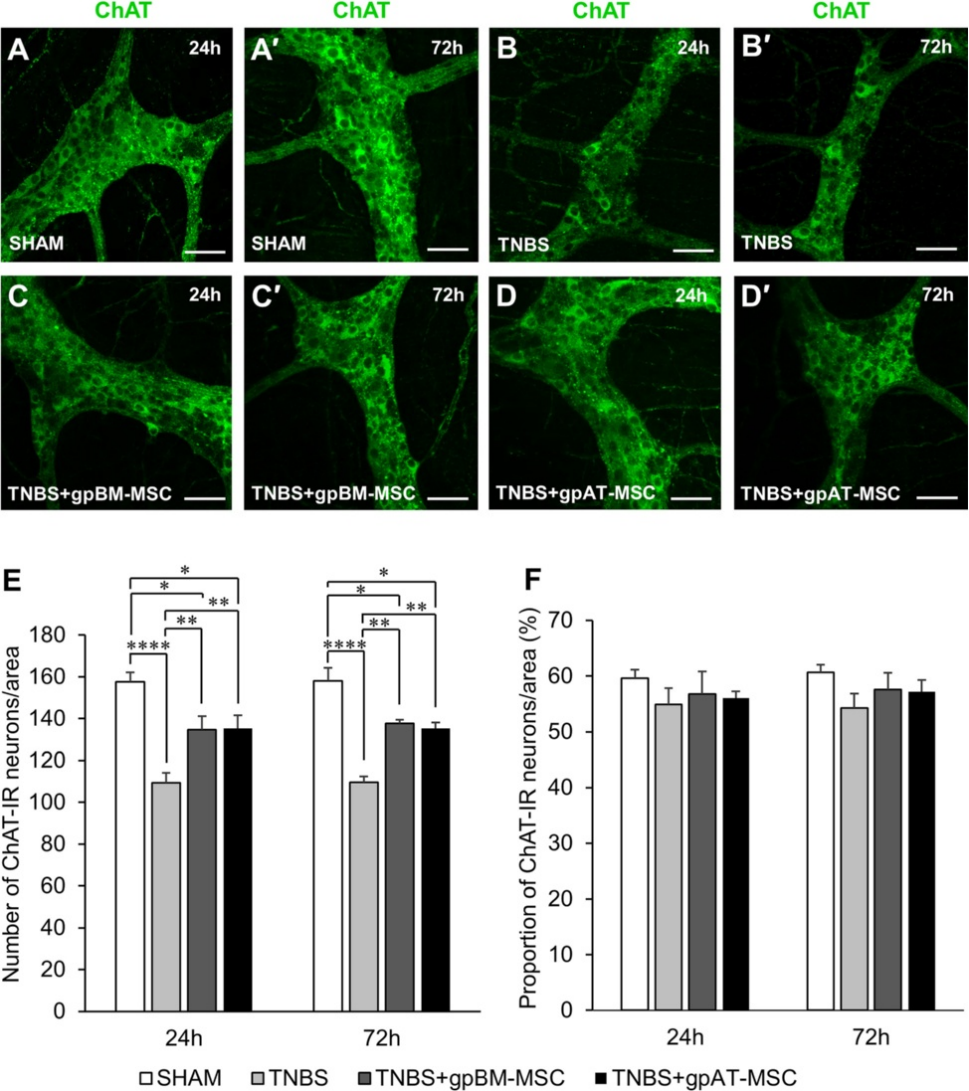
Effects of guinea pig MSCs on cholinergic myenteric neurons. **a**–*d′* Cholinergic (ChAT-IR) neurons in the myenteric plexus at 24 h (**a**–**d**) and 72 h (*a′*-*d′*). Scale bar = 50 μm. The total number of ChAT-IR neurons (**e**) and the proportion of ChAT-IR neurons to the total number of HuC/D-IR neurons (**f**) were quantified within a 2-mm^2^ area of the myenteric plexus in the guinea pig colon. *P <0.05, **P <0.01, ***P <0.001, n = 4 animals per group per time point. *gpAT-MSC* guinea pig adipose tissue-derived mesenchymal stem cell, *gpBM-MSC* guinea pig bone marrow-derived mesenchymal stem cell, *MSC* mesenchymal stem cell, *TNBS* 2,4,6-Trinitrobenzene sulfonic acid

Excitatory muscle motor and interneurons were labelled with anti-ChAT antibodies (Fig. 9a–d′). Quantification of ChAT-IR neurons revealed a decrease in the total number of neurons at both 24 and 72 h after TNBS administration compared with sham (*P <*0.0001 for both) (Fig. 9e and Table 3, n = 4 animals per group per time point). The number of ChAT-IR neurons was higher in groups treated with gpBM-MSCs and gpAT-MSCs compared with TNBS alone at both time points (*P <*0.01 for all) but was less than in shams (*P <*0.05 for all). The proportion of ChAT-IR neurons to the total number of Hu-IR neurons was quantified; however, no differences were observed between treatment groups (*P* = 0.21) (Fig. 9f).

### TGF-β1 secreted by guinea pig MSCs attenuates neuronal loss *in vitro*

**Fig. 10:**
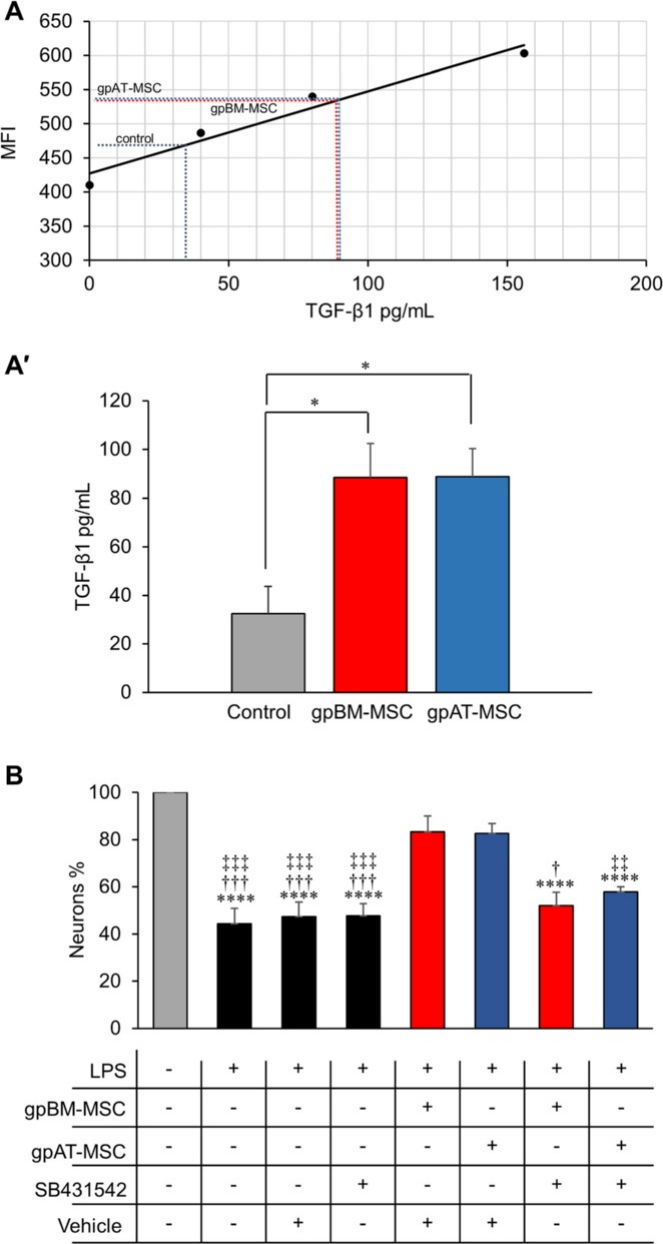
TGF-β1 secreted by guinea pig MSCs contribute to enteric neuroprotection *in vitro.* **a**, *a′* TGF-β1 secretion was assessed in gpBM-MSC and gpAT-MSC cultures. MSC-conditioned medium was collected after 48 h, and a bead-based cytometric analysis was performed. The mean fluorescence intensity (MFI) was recorded by flow cytometry, and a standard curve was generated to determine the concentration of MSC-secreted TGF-β1. Expansion medium without MSCs served as controls. *P <0.05, n = 6 independent cultures per group. **b** Primary myenteric neurons quantified within a 9-mm^2^ area. Myenteric neurons were incubated with lipopolysaccharide (LPS) to mimic inflammatory conditions. Myenteric neurons were cultured with a combination of gpBM-MSCs or gpAT-MSCs, the TGF-βR1 inhibitor SB431542 (10 μM) or dimethyl sulfoxide as a vehicle control. ****P <0.0001, significantly different from untreated myenteric neurons; †*P <*0.05, †††*P <*0.001, significantly different from LPS + gpBM-MSCs + vehicle; ‡‡*P <*0.01, ‡‡‡*P <*0.001, significantly different from LPS + gpAT-MSCs + vehicle; n = 5 independent cultures per group. *gpAT-MSC* guinea pig adipose tissue-derived mesenchymal stem cell, *gpBM-MSC* guinea pig bone marrow-derived mesenchymal stem cell, *MSC* mesenchymal stem cell, *TGF-β1* transforming growth factor-β1

TGF-β1 is a potent anti-inflammatory and neuroprotective cytokine highly conserved between species. MSCs were cultured for 48 h before the medium was collected for flow cytometric quantification of TGF-β1. The *in vitro* secretion of TGF-β1 by both gpBM-MSCs (88.4 ± 13.7 pg/ml) and gpAT-MSCs (88.7 ± 11.5 pg/ml) was observed. The expansion medium alone served as a control and also contained TGF-β1 (32.4 ± 11.2 pg/ml), albeit to a lesser extent than MSC-conditioned media (both *P <*0.05) (Fig. 10a, a′).

Primary myenteric neurons were isolated to study the role of TGF-β1 in MSC-mediated neuroprotection *in vitro* (Fig. 10b). Inflammatory conditions were simulated by incubation with LPS (100 ng/ml) for 3 h. Neuronal loss was observed in cultures incubated with LPS alone (44.4 ± 6.5 %) or in combination with the vehicle DMSO (47.3 ± 6.2 %) or the TGF-βR1 inhibitor, SB431542 (47.7 ± 5.2 %) (*P <*0.0001 for all). Neuronal loss was partly attenuated when neurons were co-cultured with gpBM-MSCs and (83.3 ± 6.6 %) gpAT-MSCs (82.5 ± 4.3 %) in comparison with LPS alone and LPS with DMSO (*P <*0.001 for all). The survival of myenteric neurons was decreased by the addition of SB431542 to co-cultures of neurons with gpBM-MSC (52.0 ± 5.6 %, *P <*0.05) or gpAT-MSC (57.8 ± 2.2 %, *P <*0.01).

## Discussion

This study is the first that has evaluated the functional capacity of guinea pig bone marrow and adipose tissue-derived MSCs and determined their therapeutic value in an animal model of colitis. *In vitro* characterisation revealed distinct differences in growth kinetics, clonogenicity and cell morphology between MSCs derived from bone marrow and adipose tissue. In an *in vivo* model of TNBS-induced colitis, gpBM-MSCs were comparatively more efficacious than gpAT-MSCs in attenuating weight loss, colonic tissue damage and leukocyte infiltration into the myenteric plexus. MSCs from both sources were equally neuroprotective in the amelioration of enteric neuronal loss and changes to the neurochemical coding of neuronal subpopulations.

MSCs were validated and characterised according to the International Society for Cellular Therapy guidelines [9]. Evaluation of surface phenotype revealed that both gpBM-MSCs and gpAT-MSCs were positive for CD29 expression. Guinea pig MSCs were negative for the expression of endothelial and haematopoietic lineage markers, CD34 and CD45. Previous use of antibodies targeting human surface marker epitopes in guinea pigs has provided evidence for cross-reactivity in CD34 and CD45 [75]; however, the possibility of false-negative results cannot be ruled out entirely. Expression of CD44 and CD90 was negligible in both types of guinea pig MSCs, and CD73 was observed only in gpBM-MSCs. The lack of CD73 expression in adipose tissue-derived MSCs has been previously reported in mouse [76]. Similar to other MSCs, gpAT-MSCs were adherent to plastic and successfully differentiated into adipocytes, osteocytes and chondrocytes when supplemented with relevant differentiation media. These traits were previously used to define cells from the guinea pig as MSCs by Frölich et al. [77] in the only other study characterising these cells. Our data demonstrated that gpAT-MSCs have greater proliferative capacity than gpBM-MSCs and this is in agreement with their study [77]. These observations are consistent with studies in mouse and human MSCs derived from bone marrow and adipose tissue [27, 78, 79].

Clonogenicity is an important characteristic of MSCs and is considered to be predictive of their proliferative and functional capacity [80]. Our data indicate that gpAT-MSCs have an approximately 13-fold higher proportion of cells able to form colonies compared with gpBM-MSCs. Decreased colony formation has been observed in human bone marrow MSCs compared with adipose tissue MSCs with CFU-f counts continuing to decline through to the fourth passage [78]. The CFU-f values of passage four guinea pig MSCs used in our study were comparable to the ranges observed by Schellenberg et al. [80] in multiple human bone marrow and adipose tissue cell lines at the same passage.

High proliferation rate and spindle-shaped morphology are desired qualities of MSCs; optimising isolation and *in vitro* conditions to maintain these traits is one of the key focuses of MSC research [81–83]. In our study, gpAT-MSCs appeared to have better MSC qualities in standard culture, including higher proliferation rate, colony-forming potential and proportions of spindle-shaped cells. Comparatively, these traits were suboptimal in gpBM-MSCs. These results may be directly related to the heterogeneous subpopulations within MSC cultures [84]. Similar to our observations in guinea pig MSCs, it has been reported that rat MSCs from adipose tissue are more spindle-shaped and have a higher proliferative capacity than those from bone marrow [85]. Given these data, characterisation of MSCs on the basis of morphology could be predictive of *in vitro* proliferation and clonogenicity. Thus, differences between the *in vitro* properties of MSCs from bone marrow and adipose tissue are regularly observed in various species. However, there is a lack of research providing comparative evaluation of the therapeutic efficacy of MSCs derived from different tissues in *in vivo* models of disease.

One of the common models of intestinal inflammation employs TNBS administration into the colon which activates the immune response mediated by T helper type 1 cells to hapten-modified autologous proteins [86]. Lack of weight gain is a symptom of TNBS-induced colitis, reflecting the general condition of the animal during the inflamed state [65, 87]. In our study, gpBM-MSC-treated, but not gpAT-MSC-treated, animals gained weight by 72 h after the induction of colitis. The absence of the cell surface enzyme CD73 (5′-nucleotidase) in gpAT-MSCs may be a contributing factor as the conversion of adenosine monophosphate to extracellular adenosine by CD73 plays a key role in suppressing the inflammatory response and the T helper type 1 pathway [88]. In our study, gpBM-MSCs alleviated gross morphological changes in histological cross-sections of the colon. Conversely, oedema was observed after gpAT-MSC treatment. Given the absence of weight gain, this may indicate that gpAT-MSCs had a reduced overall anti-inflammatory effect.

Increased infiltration of leukocytes in the mucosa, submucosa and myenteric plexus was observed after TNBS administration which was attenuated by guinea pig MSC treatments. GpBM-MSCs were more effective than gpAT-MSCs in reducing the leukocyte infiltrate into the mucosa and myenteric ganglia at 24 h after treatment. Differences between the immunomodulatory function of MSCs derived from bone marrow and adipose tissue have previously been observed *in vitro*. Human MSCs derived from bone marrow have demonstrated enhanced prevention of T lymphocyte proliferation and activation [79], whereas human adipose tissue-derived MSCs superiorly prevented monocyte-dendritic cell differentiation and promoted their release of anti-inflammatory interleukin-10 (IL-10) [89]. In contrast, an investigation into the ability of MSCs to suppress mixed lymphocyte reactions and mitogenesis found no differences between human MSCs from these tissue sources [90]. The results of *in vitro* studies are difficult to extrapolate to MSC functions *in vivo*; studies in animal models may give a better understanding of the immunomodulatory differences between MSCs from bone marrow and adipose tissue. An optimal tissue source of MSCs for the treatment of disease has not been established. Payne et al. [32] reported that human adipose tissue MSCs are more therapeutic than bone marrow MSCs in experimental autoimmune encephalomyelitis and this is presumably due to their greater migratory potential. Conversely, allogeneic MSCs derived from mouse bone marrow were observed to be more effective in the treatment of lung inflammation [91, 92]. Furthermore, human bone marrow MSCs were superior in preventing leukocyte infiltration into multiple organs in experimental systemic inflammation [93]. These seemingly polarised outcomes may suggest that the MSC source yielding optimal efficacy depends on the targeted pathology.

Administration of TNBS resulted in a loss of myenteric neurons consistent with other studies in animal models of experimental colitis [41–43]. No changes to the total number of neurons were observed between the time points of this study, demonstrating persistent neuronal loss after early inflammation. Treatment with guinea pig MSCs from both tissue sources equally alleviated neuronal loss. The neurochemical expressions of the two major subpopulations of myenteric neurons nNOS-IR (nitrergic) inhibitory and ChAT-IR (cholinergic) excitatory muscle motor and interneurons were investigated. Administration of TNBS increased the proportion and the total number of nitrergic neurons and decreased the total number of cholinergic neurons. Similar alterations to the neurochemical coding of enteric neurons have been previously reported in experimental colitis [49] and in tissues from patients with Crohn’s disease [45, 94]. Although nNOS is traditionally constitutively expressed, previous studies have also reported increased nNOS expression in neurons of the central nervous system in response to inflammatory stimuli [95, 96]. Mechanisms responsible for this have not yet been discerned. The proportion of cholinergic neurons in our study did not decrease and this is consistent with previous observations and may be attributed to the loss of total myenteric neurons [42]. Changes to the immunophenotype of myenteric neurons are associated with impaired coordination of muscular contractions and colonic dysmotility [97, 98]. Our study demonstrated that both types of guinea pig MSCs inhibited the increase in the number and proportion of nitrergic neurons and ameliorated the loss of cholinergic neurons. Similar effects were previously correlated with the attenuation of inflammation-induced colonic dysmotility by human bone marrow MSCs [68].

Although gpBM-MSCs were more effective at inhibiting leukocyte infiltration to the level of the myenteric ganglia, both gpBM-MSCs and gpAT-MSCs were equally efficient at enteric neuroprotection. This suggests that MSC-mediated neuroprotection may be directly induced by the release of neurotrophic factors. Specifically, there is increasing interest in the neuroprotective value of TGF-β1 which is secreted by MSCs [99, 100]. Our study confirmed TGF-β1 secretion in guinea pig MSC cultures; furthermore, levels of secretion were similar in MSCs from adipose tissue and bone marrow. In *in vitro* experiments, both MSCs similarly prevented myenteric neuronal loss. Furthermore, inhibition of TGF-βR1 appeared to negate the neuroprotective ability of guinea pig MSCs. TGF-β is widely regarded to have neuroprotective roles in the central nervous system for neurodegenerative diseases and ischemic insult [101, 102]. The function of the TGF-β1 isotype in neuroprotection is highlighted by Brionne et al. [103], demonstrating that neurons from TGF-β1^−/−^ mice had poor survival both *in vivo* and in culture. Furthermore, neurons from TGF-β1^−/+^ mice were vulnerable to excitotoxic injury. In human colon samples, analysis of mRNA from the myenteric plexus detected the presence of all TGF-β isoforms and receptors with high levels of TGF-β1 and TGF-βR1 [104]. TGF-β1 is known to be secreted by enteric glial cells critical to maintain neuronal survival and function [105, 106]. Recently, TGF-β secretion by MSCs was reported to be essential to elicit a therapeutic response in patients with amyotrophic lateral sclerosis [107]. Furthermore, TGF-β1 knockdown MSCs are ineffective for the treatment of ischemic brain injury in rats [108]. Thus, TGF-β may play a significant role in MSC-mediated neuroprotection of the ENS.

## Conclusions

Guinea pig MSCs are easily isolated from bone marrow and adipose tissue and exhibit characteristics similar to those observed in other species. Upon application into the guinea pig model of inflammation-induced neuropathy, allogeneic MSCs demonstrated immunomodulatory, trophic and neuroprotective properties. These data provide evidence of feasibility and functional efficacy of allogeneic MSC transplantation into guinea pig models. Linking the results of our *in vitro* and *in vivo* experiments, the gpAT-MSC profile of high proliferation rate, colony-forming potential and an increased number of cells with ‘spindle’ morphology did not provide a better therapeutic outcome. Conversely, gpBM-MSCs which were comparatively suboptimal in these *in vitro* traits exerted stronger anti-inflammatory efficacy in the treatment of plexitis *in vivo*. Both gpBM-MSCs and gpAT-MSCs had prominent neuroprotective effects alleviating neuronal loss and changes in neurochemical expression induced by intestinal inflammation. The neuroprotective action of MSCs appeared to be independent of their ability to prevent immune cell infiltrate. TGF-β1 released by both types of MSCs might have contributed to the attenuation of enteric neuropathy associated with colitis.

## Abbreviations

α-MEM: Alpha-minimum essential medium
CFU-f: Colony-forming unit-fibroblast
ChAT: Choline acetyltransferase
DMSO: Dimethyl sulfoxide
ENS: Enteric nervous system
gpAT-MSC: Guinea pig adipose tissue-derived mesenchymal stem cell
gpBM-MSC: Guinea pig bone marrow-derived mesenchymal stem cell
IBD: Inflammatory bowel disease
IR: Immunoreactive
LPS: Lipopolysaccharide
MAP-2: Microtubule-associated protein-2
MSC: Mesenchymal stem cell
nNOS: Neuronal nitric oxide synthase
OCT: Optimal cutting temperature
PBS: Phosphate-buffered saline
PDL: Population doubling level
RGB: Red, green, and blue
TGF-β1: Transforming growth factor-β1
TGF-βR1: Transforming growth factor-β receptor 1
TNBS: 2,4,6-Trinitrobenzene sulfonic acid
